# Perianal Plaques of Cytomegalovirus in a Patient with Central Nervous System Lymphoma

**DOI:** 10.1155/2012/948530

**Published:** 2012-05-17

**Authors:** Scott K. Heysell, Brian Wispelwey

**Affiliations:** Division of Infectious Diseases and International Health, University of Virginia, P.O. Box 801337, Charlottesville, VA 22908-1337, USA

## Abstract

Cutaneous manifestations of cytomegalovirus (CMV) in patients without human immunodeficiency virus remain rare. Perianal CMV may be observed due to periodic fecal shedding but may be confused for other pathogens, and definitive diagnosis requires histopathologic examination. An instructive case is described, and the literature reviewed.

## 1. Introduction

Cytomegalovirus (CMV), a member of the Herpesviridae family of DNA viruses, is an important pathogen in the immunocompromised host. The patterns of organ involvement and clinical disease can be characteristic in particular populations, such as gastrointestinal ulceration and hepatitis following solid organ transplant, retinitis in patients with acquired immunodeficiency syndrome (AIDS), and myelosuppression and pneumonitis in bone marrow transplant patients [1–3]. Cutaneous manifestations, however, particularly in patients without AIDS, remain rare. We discuss a case of CMV presenting as perianal plaques in a patient with central nervous system lymphoma and highlight the importance of prompt histopathologic diagnosis and early antiviral therapy.

## 2. Case Report

**Figure 1:**
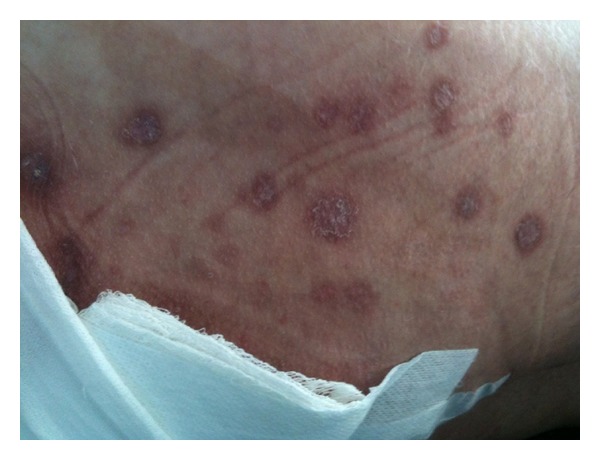
Perianal plaques in various stages of eruption.

A 47 year-old-man, human immunodeficiency virus negative, with a recent diagnosis of central nervous system lymphoma presented with perianal skin lesions and nonbloody diarrhea. The skin lesions began as nodules that progressed to a roughened plaque and in the minority, ulceration. The lesions began eight weeks prior to presentation, were multiple, scattered in the perianal area and upper thigh bilaterally, and three centimeters at the widest diameter (Figure 1). A more distinct lesion with irregular border was present over the sacrum consistent with a decubitus ulcer. The patient had been diagnosed with a CNS lymphoma six weeks prior to admission when he presented to another hospital with lower extremity weakness, saddle anesthesia, and fecal incontinence, and was found to have a L1-L2 intramedullary spinal mass. He had emergent surgical resection of the mass and was treated with high-dose dexamethasone and further radiation therapy. He was additionally found to have a right temporal lobe mass that was resected and pathology was consistent with a B-cell lymphoma with T-cell infiltration. He had no other significant past medical history or immunocompromising condition. The lower extremity weakness persisted and primarily confined him to bed. For ongoing fecal incontinence and the nonhealing sacral decubitus ulcer, he ultimately had a diverting colostomy. Two weeks prior to presentation, his bowel movements became more liquid in consistency and increased in frequency. He denied abdominal pain. The perianal skin lesions were not painful, but the initial loss of sensation from the compressive spinal mass had not recovered. He remained on a dexamethasone taper and was transferred to this hospital for further evaluation for chemotherapy. His complete blood count was notable for a normocytic anemia, and his liver function tests for an elevated alkaline phosphatase. An excisional biopsy of one of the skin lesions from the upper thigh was obtained and sent for pathological examination.

**Figure 2:**
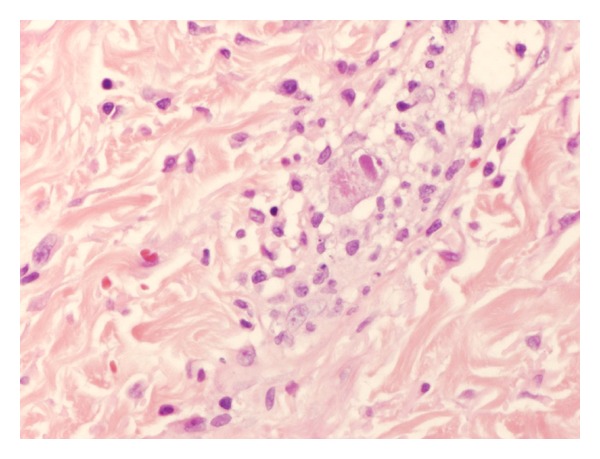
Skin biopsy with hematoxylin/eosin at 400x power. Lymphocytic infiltrate of dermis with enlarged endothelial cells and occasional magenta-colored intranuclear inclusions.

**Figure 3:**
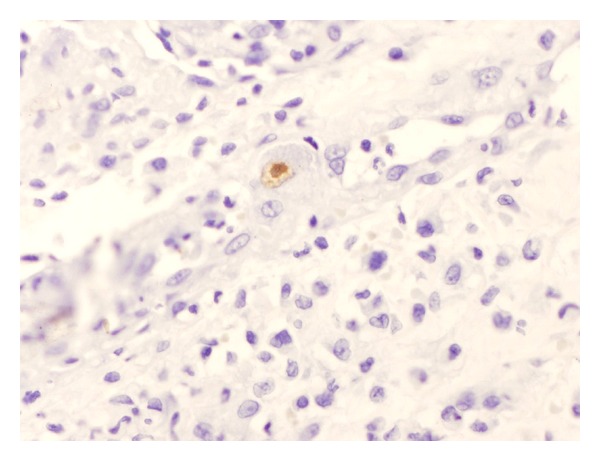
Skin biopsy with immunoperoxidase stain at 400x power. Cytomegalovirus positive cell featured in the center of view.

Sections of the pathological specimen revealed a dense neutrophilic crust with numerous bacteria distributed in varying sized aggregates, and the underlying epidermis was acanthotic with artefactual subepidermal clefting. However, the superficial dermis demonstrated a distinct perivascular lymphocytic infiltrate, and the endothelial cells were markedly enlarged (Figure 2). Some endothelial cells contained magenta-colored nuclear inclusions consistent with cytomegalovirus (CMV) infection which was confirmed by a positive CMV immunostain (Figure 3). Stains with periodic acid-Schiff and for acid-fast bacilli were negative.

The patient was treated with intravenous ganciclovir at five mg/kg every twelve hours for 21 days followed by transition to valganciclovir. A serum CMV viral load was 16,400 copies/mL at treatment initiation and was undetectable after four weeks of treatment. The diarrhea resolved within two weeks, and the skin lesions had completely healed at the time of switch to valganciclovir. Based on pathological findings and response to treatment, the diagnosis of cutaneous CMV was made. The primary mode of pathogenesis was thought to be local inoculation from fecal shedding. The diarrhea appeared to be a manifestation of CMV colitis, but given the commitment to CMV treatment and the response to therapy, no further investigation was performed. Unfortunately, the patient later died secondary to complications of an intracranial hemorrhage at the site of the temporal lobe resection.

## 3. Discussion

Cutaneous CMV infection is thought to arise from reactivation of latent virus in endothelial cells, by endothelial colonization in the course of hematogenous dissemination, or by autoinoculation in perioral or perianal areas by fecal, urinary or salivary shedding of CMV at the time of immunosuppression. The predominant defense against CMV is the MHC-restricted cytotoxic T cell, with growing evidence for a contributory role played by CD4^+^T cells and humoral immunity [4]. Certain immunosuppressive regimens such as induction with antithymocyte globulin are associated with TNF-*α*-induced CMV reactivation. Furthermore, maintenance therapy with cyclosporine, tacrolimus, and prednisone can suppress cytotoxic cells in a dose-dependent manner. Earlier reports suggested that the dermis was not conducive to CMV replication, and that cutaneous manifestation was only a marker of disseminated disease in a severely immunocompromised host. Furthermore, CMV may periodically colonize perianal decubiti without histopathologic change. Therefore, definitive diagnosis requires histology marked by typical vascular endothelial cytopathic changes; intracytoplasmic and intranuclear inclusions affecting capillaries. Interestingly, histological CMV neuritis has been described in perianal ulcerations with targeted biopsy and may explain pain similar to that of varicella zoster [5].

A variety of cutaneous manifestations of CMV have been described in patients with HIV/AIDS, yet most cases were concurrently infected with herpes simplex virus. The majority of lesions found in the perianal area have been described as ulcers and plaques, yet nodules and maculopapular eruptions, particularly when found elsewhere on the body have been reported [6], and in severe cases, necrosis has also been described. Ulcers may be single or multiple and vary in size with as large as 15 cm documented [5]. Like herpes simplex, pain or pruritis is common but not requisite.

Prior to the availability of ganciclovir, cutaneous CMV often portended death and diagnosis was frequently made postmortem. Case series in non-HIV/AIDS patients describe excellent clinical response to ganciclovir and reduction in immunosuppression [6], and this is particularly true for patients in which antigenemia or serum viral load-based diagnosis precedes biopsy. There are no trials of treatment for cutaneous CMV, but duration has been extrapolated from that of infection in other organs where 14–21 days of induction with ganciclovir may be adequate [3]. Appropriately treated lesions still may require 1-2 months until complete resolution [5]. As serum levels of valganciclovir are equivalent to ganciclovir, it is no surprise that case reports exist of successful cure with valganciclovir alone [7].

This case highlights the importance of increased suspicion for cutaneous CMV in patients with underlying immunosuppression, particularly in areas of the skin prone to autoinoculation. Diagnosis is based on definitive histopathology which may not be widespread, and thus one must maintain a low threshold for excisional biopsy.
